# Everything You Always Wanted to Know about Sarcopenia but Were Afraid to Ask: A Quick Guide for Radiation Oncologists (impAct oF saRcopeniA In raDiotherapy: The AFRAID Project)

**DOI:** 10.3390/curroncol29110671

**Published:** 2022-11-08

**Authors:** Federica Medici, Stefania Rizzo, Milly Buwenge, Alessandra Arcelli, Martina Ferioli, Gabriella Macchia, Francesco Deodato, Savino Cilla, Pierandrea De Iaco, Anna Myriam Perrone, Silvia Strolin, Lidia Strigari, Gloria Ravegnini, Alberto Bazzocchi, Alessio G. Morganti

**Affiliations:** 1 Department of Experimental, Radiation Oncology, Diagnostic and Specialty Medicine-DIMES, Alma Mater Studiorum University of Bologna, Via Albertoni 15, 40138 Bologna, Italy; 2 Service of Radiology, Imaging Institute of Southern Switzerland, Ente Ospedaliero Cantonale (EOC), 6900 Lugano, Switzerland; 3 Radiation Oncology, IRCCS Azienda Ospedaliero-Universitaria di Bologna, 40138 Bologna, Italy; 4 Radiation Oncology Unit, Gemelli Molise Hospital-Università Cattolica del Sacro Cuore, 86100 Campobasso, Italy; 5 Medical Physics Unit, Gemelli Molise Hospital-Università Cattolica del Sacro Cuore, 86100 Campobasso, Italy; 6 Division of Oncologic Gynecology, IRCCS Azienda Ospedaliero-Universitaria di Bologna, 40138 Bologna, Italy; 7 Centro di Studio e Ricerca delle Neoplasie Ginecologiche (CSR), University of Bologna, 40138 Bologna, Italy; 8 Department of Medical Physics, IRCCS Azienda Ospedaliero-Universitaria di Bologna, 40138 Bologna, Italy; 9 Department of Pharmacy and Biotechnology, University of Bologna, 40126 Bologna, Italy; 10 Diagnostic and Interventional Radiology, IRCCS Istituto Ortopedico Rizzoli, 40136 Bologna, Italy

**Keywords:** literature review, narrative review, radiotherapy, sarcopenia, adult cancer, pediatric cancer, prognostic factors

## Abstract

Sarcopenia (SP) is a syndrome characterized by age-associated loss of skeletal muscle mass and function. SP worsens both acute and late radiation-induced toxicity, prognosis, and quality of life. Myosteatosis is a pathological infiltration of muscle tissue by adipose tissue which often precedes SP and has a proven correlation with prognosis in cancer patients. Sarcopenic obesity is considered a “hidden form” of SP (due to large fat mass) and is independently related to higher mortality and worse complications after surgery and systemic treatments with worse prognostic impact compared to SP alone. The evaluation of SP is commonly based on CT images at the level of the middle of the third lumbar vertebra. On this scan, all muscle structures are contoured and then the outlined surface area is calculated. Several studies reported a negative impact of SP on overall survival in patients undergoing RT for tumors of the head and neck, esophagus, rectum, pancreas, cervix, and lung. Furthermore, several appetite-reducing side effects of RT, along with more complex radiation-induced mechanisms, can lead to SP through, but not limited to, reduced nutrition. In particular, in pediatric patients, total body irradiation was associated with the onset of SP and other changes in body composition leading to an increased risk of cardiometabolic morbidity in surviving adults. Finally, some preliminary studies showed the possibility of effectively treating SP and preventing the worsening of SP during RT. Future studies should be able to provide information on how to prevent and manage SP before, during, or after RT, in both adult and pediatric patients.

## 1. Introduction

Sarcopenia (SP) is a syndrome characterized by loss of skeletal muscle mass (quantitative impairment) and function (qualitative impairment) [1,2,3]. In recent years, interest in SP in oncology has been growing due to the high prevalence of SP (15–74%) in cancer patients [4].

In particular, attention to SP has grown in the field of radiotherapy (RT), given the evidence of the significant impact of SP on the prognosis of RT-treated patients, and the possibility of preventing and at least partially treating this syndrome [5]. In fact, only in 2020 and 2021, 114 papers were registered on PubMed including seven systematic reviews on RT and SP [6]. For this reason, it may be useful to provide a quick guide to radiation oncologists on this topic.

Therefore, the aim of this narrative review is to offer some brief information regarding pathophysiology, screening, diagnosis, prognostic impact, and management of SP in RT-treated adult and pediatric patients, by providing answers to “possibly” frequently asked questions. The review was written by a multidisciplinary team consisting of radiation oncologists, radiologists, health physicists, oncology surgeons, and translational cancer research experts. The rationale and concept for this manuscript were proposed and discussed by the authors during a videoconference in January 2022. This narrative review is part of the AFRAID (impAct oF saRcopeniA In raDiotherapy) project.

## 2. General Definitions and Concepts

**What is sarcopenia? How is it defined?**

Three consensus statements [1,2,3] agreed to define SP as a syndrome characterized by age-associated loss of skeletal muscle mass (quantitative impairment) and function (qualitative impairment) combined or not with increased fat mass (**sarcopenic obesity**—SO). Moreover, the European consensus separated **primary** (no etiology found) and **secondary SP** (associated with physical inactivity or some chronic conditions: malnutrition, endocrinopathies, chronic diseases, inflammatory disease, or cancer) [1]. Furthermore, **pre-SP** is considered an isolated loss of skeletal muscle without impaired muscle function [1]. Finally, in cancer patients, SP is considered the first step toward **cachexia**, a condition that is not fully reversible with nutritional intervention and leads to disability and reduced treatment efficacy.

**What are the causes and mechanisms leading to sarcopenia?**

**Figure 1:**
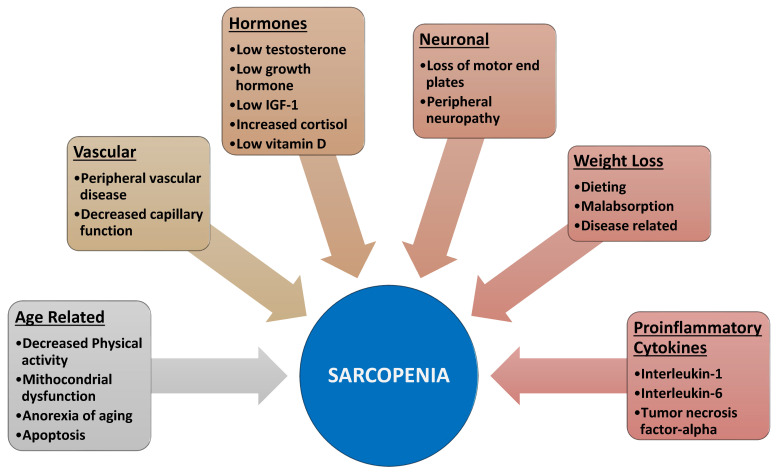
Physiopathological processes of sarcopenia (from Morley JE, et al. 2014, modified [15]).

The origin of SP is considered to be multifactorial (Figure 1). In particular, the following are considered causes of SP:

1 **Aging** is associated with a state of anorexia leading to weight loss [7,8]. The latter is due to both a reduction in fat (75%) and muscle and bone tissue (25%). In the case of weight recovery, this mainly affects the fat tissue leading to SO.
2 Furthermore, aging is also associated with **motor neuron alterations** [9] leading to muscle atrophy and decreased muscle function.
3 In the elderly **anabolic hormones** (growth hormone, insulin growth factor 1, DHEA, and testosterone) are reduced [10,11,12].
4 Aging and obesity favor a condition of **insulin resistance**, leading to reduced availability of glucose and proteins needed for muscle anabolism [13].
5 Finally, obesity and various diseases increase **proinflammatory cytokines** and thus activate NFkB and ultimately protein catabolism [14].

**What are the clinical, biochemical, and molecular mechanisms underlying reduced therapeutic response?**

Some mechanisms explaining the negative prognostic impact of SP in oncology can be summarized as follows:

1 SP is closely related to **performance status,** which in turn has an important and well-known prognostic impact on cancer patients [16,17].
2 SP is associated with a higher incidence of **peri- and post-operative complications** and therefore can delay or hinder adjuvant therapies with consequently worse prognosis [18,19].
3 The loss of muscle mass reduces the secretion of some **circulating cytokines,** which are produced by muscle cells (myokines: IL-6, IL-8, IL-15) and which hinder tumor progression.
4 SP is more common in patients with **more advanced cancer** [18,20,21].
5 SP worsens the frequency and severity of radiation-induced **acute toxicity** [22,23] and thus hinders the completion of RT on schedule [24].
6 SP is associated with increased radiation-induced **late toxicity**, with possible worsening of prognosis and quality of life [25].

Overall, SP is closely related to the patient’s general condition and can affect both tumor progression and treatment tolerability [4].

**Why is sarcopenia different from cachexia and myosteatosis?**

Based on an international consensus, cachexia was defined as the progressive and not completely reversible loss of skeletal muscle tissue associated with impaired muscle function. In particular, cachexia is considered to be a syndrome including successive stages, from pre-cachexia to cachexia, to refractory cachexia. Cachectic patients are subjects who have shown a weight reduction > 5% in six months or patients who have shown a weight reduction of at least 2% and concurrently have a BMI < 20 kg/m^2^ or an SP status [26]. Cachexia is caused by the combination of reduced dietary intake and alterations in metabolism and in particular, as SP, to the activation of factors regulating the degradation of proteins, even if the type of mediators is different from those involved in SP [27]. More specifically, SP appears to be related to changes in signals for muscle tissue growth, whereas cachexia would depend on cytokine-mediated degradation of muscle and adipose tissue [28]. Furthermore, cachexia, as well as the state of systemic inflammation, seem to be associated with the activation of specific genes that are more expressed in some tumors (lung, pancreas) in which the cachectic state is more frequent [29]. A more accurate understanding of the biomolecular pathways involved in SP and cachexia could lead to the development of drug therapies targeting these processes.

Myosteatosis (MS) is a pathological infiltration of muscle tissue by adipose tissue. In fact, in physiological conditions, skeletal muscles contain only minimal fat deposits which, during aerobic activity, are used as a source of energy. MS increases in old age and negatively affects metabolism as it is associated with insulin resistance and diabetes. The deposition of adipose tissue in the muscles can occur in different ways/sites:

1 Between different muscles (intermuscular),
2 In the extracellular site but within a single muscle (intramuscular),
3 Within the cells (intramiocellular). Furthermore, MS is characterized not only by the accumulation but also by the different chemical compositions of fats normally present in the muscles [30].

Therefore, MS is not synonymous with SP but the two conditions can coexist and produce synergistic effects, particularly at the metabolic level [31]. Indeed, MS is associated with diabetes and obesity [32,33,34], impaired muscle function [35], and cancer [36]. Its correlation with the prognosis in cancer patients has also been demonstrated [37].

**What is sarcopenic obesity? Is it worse than Sarcopenia? Why?**

SO is considered a “hidden form” of SP (due to large fat mass) [38]. Due to the lack of a shared definition, the prevalence varies between the different studies [38]. SO is independently related to higher mortality and worse complications after surgery and systemic treatments, with a worse impact compared to SP alone. The reasons for this effect are not fully understood but the simplest explanation is that patients with SO have both the risks of obesity and SP [39]. Furthermore, patients with SO are typically unfit and unable to tolerate stressful situations [38].

Moreover, it has been speculated that the greater chemotherapy toxicity in patients with SO is due to a high absolute drug dose which is distributed in a small volume. In fact, the Body Surface Area is traditionally used to calculate the dose of cytotoxic chemotherapy. Nevertheless, in patients with SO, the large Body Surface area determines a high dose of chemotherapy but is distributed in a reduced lean body mass with consequently hindered metabolism and elimination of drugs, and thus with a higher incidence of toxicity [38].

The clinical management of SO requires further study, starting with the formulation of a consensus definition. In fact, the following appear to be particularly necessary: (I) pharmacology studies to investigate the effect of SO on the distribution, metabolism, and elimination of chemotherapeutics, (II) analyses confirming the synergistic effect of PD and obesity, (III) studies aimed at defining SO treatment protocols and methods of dose-modulation of systemic therapies in subjects with SO. Indeed, at present, the only treatment strategy in this setting is empirically based on the combination of weight loss, adequate protein intake, and exercise [38,40].

## 3. Detection of Sarcopenia

**How to screen and diagnose sarcopenia?**

The definition of SP has evolved over the last few years and several tools have been proposed to screen for this condition (Table 1).

The most frequently used parameters were “skeletal muscle mass”, “appendicular skeletal muscle mass”, and muscle cross-sectional area at specific body sites [41]. However, these parameters are affected by body size and therefore need to be corrected taking into account the value of the latter. This correction was performed using height, weight, body mass index, or body surface area [50]. Regardless of the parameters used to identify patients with SP, subjects with values less than two standard deviations from the values measured in young and healthy adults were generally defined as sarcopenic [41,50].

A particularly simple tool for SP screening is the **SARC-F**, a five-items questionnaire for patients [42]. The European consensus on the definition and diagnosis of SP proposed more complex diagnostic assessments based on the quality and quantity of muscles, muscle strength, and general physical performance. The use of these criteria allows one to distinguish suspected from confirmed and serious sarcopenic subjects [41].

**Figure 2:**
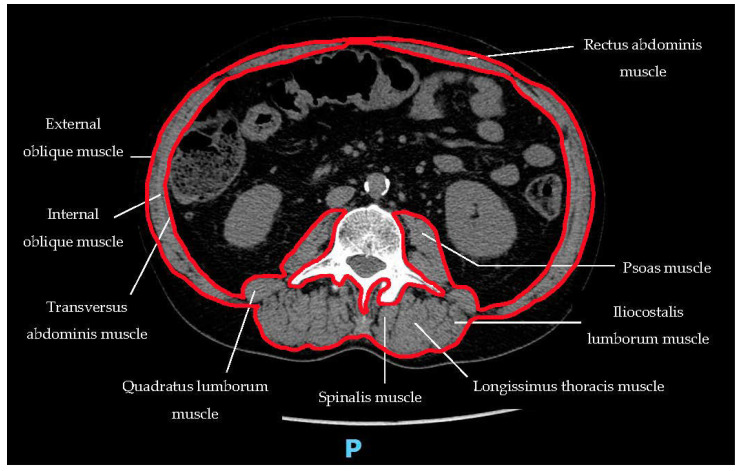
Example of delineation of the skeletal muscle area on a CT scan at the level of the third lumbar vertebra.

Instead, instrumental evaluation of SP is commonly based on CT images. In particular, a scan at the level of the middle of the third lumbar vertebra (L3) is generally used [51], based on the strong correlation between muscle “surface” at this level and whole-body muscle volume [52]. On this scan, all muscle structures are contoured (Figure 2) and then the outlined surface area (defined as: **skeletal muscle area**—SMA) is calculated. Based on this method, we defined as sarcopenic the subjects with **skeletal muscle index** (SMI: SMA divided by body surface area) <52.4 cm^2^/m^2^ and <38.5 cm^2^/m^2^ in men and women, respectively, based on the classic criteria from Prado et al. [53]. However, different cut-off values have been proposed for use in non-European or North American populations (Bas 2019). Moreover, the Prado et al. criteria were partially corrected by Martin et al., as shown in Table 2 [54].

As reported above, CT is the preferred method to identify SP patients due to its accuracy in assessing both the quantity and density of muscles [51,54]. Regarding the anatomical level of the CT scan, the recommended choice is L3, as previously reported [56]. However, evaluations performed in other anatomical sites, in particular at the third cervical vertebra, showed a close correlation with the values measured at L3. The use of a contrast medium during the CT scan does not affect the assessment of muscle mass but can influence the assessment of muscle density. Therefore, it was suggested to acquire the images for the study of density in the portal phase of the CT scan [57]. In present times, some advanced software allow us to automatically extract from CT scans not only the SMA values but also other parameters on body composition [58].

## 4. Impact of Sarcopenia

**What is the impact of sarcopenia in patients treated with radiotherapy?**

Most of the current evidence on the impact of SP in patients undergoing RT comes from analyses on patients with head and neck tumors [5,59]. However, several studies reported a significantly negative impact of SP on overall survival not only in patients undergoing RT for head and neck cancers [25,60,61,62,63], but also in the esophagus [64], rectum [65], pancreas [66], cervix [67], and lung [68] tumors.

In addition, significantly worse toxicity was observed in patients with SP and treated with RT for head and neck tumors [25,63]. This result led to the hypothesis that the negative impact of outcome derives at least in part from poor tolerance and frequent RT breaks in sarcopenic subjects [63]. Furthermore, a negative impact has been reported in patients with SP and undergoing RT, both on local control and on metastasis-free survival [60].

Finally, several studies reported that, by analyzing SP together with other traditional prognostic factors, the impact of the SP was independently significant [25,60,64].

**What is the impact of radiotherapy on sarcopenia?**

Whether and to what extent RT induces the development of SP is an important problem but at the moment has been little studied, despite the fact that markers and tools have been proposed to monitor RT-induced nutritional disorders [59]. In general, it is believed that several side effects of RT (nausea, diarrhea, esophagitis, taste alterations, xerostomia) negatively impact appetite and therefore lead to SP through reduced nutrition. To confirm this, a prospective study on patients irradiated with tumors in various body sites showed that all subjects had a reduction in BMI that reached the maximum values at the end of treatment, with changes in body composition varying according to the type of tumor and irradiated body site [69]. However, other evidence shows a more complex picture than the simple causal correlation between toxicity and malnutrition. In fact, a study conducted on patients with non-small cell lung cancer undergoing RT and chemotherapy (concurrent or sequential) showed an early weight reduction (especially in patients undergoing concurrent therapy) independent of the onset of radiation-induced esophagitis and nutritional intake [70].

**What is the impact of sarcopenic obesity and myosteatosis on radiotherapy outcomes?**

In the report of Ahern et al., the negative impact of SP and MS on overall survival was stronger compared to the one of SP alone [71]. Furthermore, in Findlay et al. study on the impact of SP and MS in patients undergoing curative RT for head and neck cancer, post-treatment SP and pre-treatment MS were independent predictors of reduced overall survival at multivariable analysis [60]. Moreover, MS was independently associated with postoperative morbidity and adverse oncologic outcomes in patients with retroperitoneal and trunk sarcoma treated with preoperative RT and surgery [72]. On the contrary, Findlay et al., in their study on patients with head and neck cancer treated with RT, reported that malnutrition (evaluated using the Scored Patient-Generated Subjective Global Assessment) but not SP and MS was independently associated with reduced overall survival at multivariable analysis [61].

In the report of Li et al., SO (and not SP alone) independently predicts survival in patients with gastric cancer treated with adjuvant chemoradiation [73]. Finally, Stangl-Kremser et al. assessed the prognostic impact of SP in patients with bladder cancer treated with RT alone and reported that SO was significantly associated with cancer-specific survival [74].

**Can sarcopenia impact treatment modulation in patients who are candidates for radiotherapy?**

The possibility of modulating RT based on the presence of SP would clearly be very useful. In fact, theoretically, it would be useful to escalate dose, fractionation, and concurrent and adjuvant therapies in patients with a lower probability of local control, and to de-escalate treatment in patients with a higher risk of serious toxicities that could preclude RT completion. More generally, it would be useful to modulate the treatment in order to improve the efficacy and tolerability of RT in patients with SP.

However, these strategies have only been suggested [63,75,76,77], but never tested in clinical trials.

**Can we improve the prognostic impact of sarcopenia by including it in predictive models?**

Given the current interest in the development of predictive models to individualize cancer treatments, and in particular RT, the design of models including SP along with other prognostic factors seems an inevitable development of research on this issue. However, there are currently only sporadic reports on this topic [77,78,79].

## 5. Management of Sarcopenia during Radiotherapy

**Is it possible to prevent sarcopenia before radiotherapy?**

This is obviously an important problem considering both the negative impact of RT on nutritional status and the negative impact of SP on outcomes after RT. Therefore, a pre-RT assessment of baseline levels would be desirable to identify early impairments or predisposing factors of the latter and to provide support to prevent subsequent deterioration of body composition and functional status. Unfortunately, this topic has also been little studied. In a pilot study in patients with esophageal tumors undergoing neoadjuvant chemoradiation, a health coaching mobile app (Noom) was tested to advise patients on exercise and dietary intake. However, the intervention was not able to prevent the onset of SP and the authors concluded that more complex and personalized interventions seem necessary at least in that setting [80].

**Is it possible to treat sarcopenia before and during radiotherapy?**

Two systematic reviews of the literature have addressed this topic. The authors of the first analysis evaluated the relationship between protein intake and maintenance of muscle mass in patients undergoing RT for head-neck, lung, or esophageal cancers. The results of the study showed that the intake necessary to avoid the loss of muscle mass during RT is higher than the usual recommendations (>1.4 g/kg vs. 1.2 g/kg) [76]. The authors of the second review analyzed the impact of nutritional interventions and exercise-based interventions in patients undergoing RT for head and neck cancers. The authors concluded that both interventions are useful for maintaining nutritional status and physical functions while, surprisingly, the combination of the two interventions was not effective [75]. More studies are needed on this topic, considering the possibility of improving the nutritional status of cancer patients through increased intake of calories and proteins and the use of immunological response modulators [81].

**Should we screen for and treat sarcopenia in radiation-treated pediatric patients too?**

Only a few studies are available on this topic. In particular, two studies evaluated the incidence of SP, and more generally of metabolic problems, in pediatric patients with leukemia treated with hematopoietic stem cell transplant (HSCT) and total body irradiation (TBI) [82].

Wei et al. analyzed a population of survivors of childhood acute lymphoblastic leukemia treated with HSCT and TBI. These patients were compared with a group of subjects who had received only chemotherapy and with a group of non-leukemic but obese subjects. Compared to the other groups, patients undergoing HSCT and TBI had lower insulin sensitivity, increased distribution of visceral, intramuscular, and total fat and, together, reduced lean body mass [82].

Nakayama et al., in a similar analysis on adult survivors of childhood leukemia/lymphoma, reported higher risk of developing SP after HSCT and of developing obesity after cranial radiotherapy [83].

Recently, Lorenc et al. published a systematic review of body composition changes in patients 0 to 24 years of age undergoing HSCT plus TBI. This literature review showed that a lipodystrophic phenotype and loss of skeletal muscle mass are common in these patients. The authors concluded that interventions aimed at improving fat and muscle function should be tested in these subjects, possibly through nutritional manipulations or physical activity [84].

An attempt in this direction has been initiated and is currently being explored by the group of Song and colleagues. These authors reported promising results, in the prevention of post-HSCT SP, from the combination of exercise plus exercise enhancers such as nicotinamide riboside (NR) and a nicotinamide adenine nucleotide (NAD+) precursor. In fact, these two factors are able to increase mitochondrial oxidative phosphorylation and thus muscle function and strength [85].

Other authors also explored the feasibility and efficacy of preventive interventions aimed at counteracting the change in body composition observed after HSCT, despite the unchanged values of BMI and waist circumference [86].

Rupnik et al. investigated the feasibility of a multimodal intervention program based on the combination of exercise (aerobic 4 days/week and resistance exercise 3 days/week) and nutritional support in 34 HSCT candidate patients enrolled at least 2 weeks before treatment start [87]. Davis et al. tested a 6-month aerobic and resistance exercise program in patients already undergoing HSCT/TBI [88].

In both studies, a measurable improvement in patients’ physical performance and QoL was reported.

## 6. Future Studies

**Which future studies on sarcopenia in the radiotherapy setting are needed?**

First, and not only in the RT setting, a broader consensus on definitions and cut-offs is needed to better identify patients with SP, SO, MS, and cachexia. This is a necessary prerequisite for using body composition information as a prognostic and predictive tool in clinical practice. Moreover, it would be useful to test innovative and non-invasive methods for screening SP, capable of replacing or supplementing those currently in use. For example, the D3-Creatine dilution test, based on the enrichment of D3-creatinine in a single urine sample, appears to be a promising method for evaluating total body creatine pool size and thus skeletal muscle mass [89]. Furthermore, studies are needed to define the best strategies for the prophylaxis and treatment of SP, in terms of diet and exercise, possibly individualizing them according to the specific patient’s characteristics. Then, any treatment strategy has to be tested and compared in randomized controlled trials.

Moreover, investigations on the effect of SO on the distribution, metabolism, and elimination of chemotherapeutics and further analyses confirming the synergistic effect of SP and obesity are needed, as well as studies aimed at defining methods of dose-modulation of systemic therapies in subjects with SO.

In particular, in the RT setting, future studies should be able to provide information on how to prevent and manage SP before, during, or after RT. Furthermore, they should provide guidance on how to modulate RT or chemoradiation based on the presence and severity of SP. Finally, considering that MS generally appears before SP, the role of MS as an early prognostic factor in patients undergoing RT should be investigated.

## 7. Conclusions

A summary of the topics of this review is provided in Table 3.

Greater awareness of the importance of SP in RT-treated patients is needed, considering the impact on toxicity and oncological outcomes. Moreover, future studies are needed to define the optimal tools for SP screening and diagnosis and especially to identify the most effective therapies to prevent and treat SP before, during, and after RT, with the aim to enhance rehabilitation and limit progression toward cachexia and disability. In this regard, circulating (microRNA) and tissue-specific factors may represent both potential biomarkers and therapeutic targets of SP.

## Figures and Tables

**Table 1:** Tools to screen sarcopenia in clinical practice (From Cruz-Jentoft AJ et al., adapted [41]).

Variable	Tool (Reference)
*Case finding*	*SARC-F questionnaire* [42] *Ishii screening tool* [43]
*Skeletal muscle strength*	*Grip strength* [44]; *Chair stand test* (chair rise test) (American Academy of Orthotists & Prosthetists) [45]
*Skeletal muscle mass or skeletal muscle quality*	Appendicular skeletal muscle mass by *Dual-energy X-ray absorptiometry* [46]; Whole-body skeletal muscle mass or appendicular skeletal muscle mass predicted by *Bioelectrical impedance analysis* [47]; *Lumbar muscle cross-sectional area* by CT or MRI [48,49]
*Physical performance*	*Gait speed* (NIH Toolbox 4), *Meter Walk Gait Speed Test* (https://www.nia.nih.gov/research/labs/leps/short-physical-performance-battery-sppb) °; *Short physical performance battery* (Short Physical Performance Battery Protocol, https://research.ndorms.ox.ac.uk/prove/documents/assessors/outcomeMeasures/SPPB_Protocol.pdf); *Timed-up-and-go test* [43]; *400-m walk or long-distance corridor walk* [44]

° Short Physical Performance Battery (SPPB). National Institute on Aging. https://www.nia.nih.gov/research/labs/leps/short-physical-performance-battery-sppb (accessed on 29 July 2022).

**Table 2:** Common cut-offs for sarcopenia definition based on CT-scan at the level of the third lumbar vertebra.

	Skeletal Muscle Index (SMI)			Skeletal Muscle Mean Radiation Attenuation (SMRA) *
	**Women**	**Man**		
Prado et al. [53]	≤38.5 cm^2^/m^2^	≤52.4 cm^2^/m^2^		
Martin et al. [55]	<41 cm^2^/m^2^	<43 cm^2^/m^2^ if BMI < 25	<53 cm^2^/m^2^ if BMI ≥ 25	
Van der Werf et al. [48]				<29 Hounsfield Units

* Non-contrast-enhanced single-energy CT scans

**Table 3:** Summary of questions and answers.

Questions	Answers
What is sarcopenia? How is it defined?	Sarcopenia is a syndrome characterized by age-associated loss of skeletal muscle mass (quantitative impairment) and function (qualitative impairment) combined or not with increased fat mass (sarcopenic obesity).
What are the causes and mechanisms leading to sarcopenia?	Main causes/mechanisms are: ○ Aging-associated anorexia leading to weight loss. ○ Aging-associated motor neuron alterations leading to muscle atrophy. ○ Aging-related anabolic hormones reduction. ○ Insulin resistance leading to reduced availability of glucose and proteins for muscle anabolism. ○ Obesity-related pro-inflammatory cytokines and activating protein catabolism.
What are the clinical, biochemical, and molecular mechanisms underlying reduced therapeutic response?	**Sarcopenia** is closely related to: ○ Worse performance status, ○ Higher incidence of peri- and post-operative complications, and thus ○ Delayed or hindered adjuvant therapies, ○ Reduction of circulating cytokines hindering tumor progression, ○ More advanced cancer stage, ○ Worsened acute and late radiation-induced toxicity.
Why is sarcopenia different from cachexia and myosteatosis?	**Cachexia:** ○ Is a multiphasic syndrome (from pre-cachexia, to cachexia, to refractory cachexia) with progressive and not completely reversible loss of skeletal muscle tissue associated with impaired muscle function. ○ Is produced by the combination of reduced dietary intake and alterations in metabolism with activation of factors regulating the degradation of proteins. ○ Is associated with the activation of specific genes that are more expressed in some tumors (lung, pancreas). **Myosteatosis:** ○ Is a pathological infiltration of muscle tissue by adipose tissue, increases in older age, and negatively affects metabolism. ○ Can be intermuscular, intramuscular, and intramiocellular. ○ Can coexist with sarcopenia producing synergistic effects. ○ Is associated with diabetes, obesity, impaired muscle function, and cancer ○ Is correlated with worse prognosis in cancer patients.
What is sarcopenic obesity? Is it worse than sarcopenia? Why?	**Sarcopenic obesity:** ○ Is as a “hidden form” of sarcopenia (due to large fat mass) ○ Is independently related to higher mortality and worse complications after surgery and systemic treatments, with a worse impact compared to sarcopenia alone due to combination of the risks of obesity and sarcopenia. ○ In patients with sarcopenic obesity, the large body surface area determines a high dose of chemotherapy but distributed in a reduced lean body mass, with higher incidence of toxicity.
How to screen and diagnose sarcopenia?	The screening of sarcopenia is based on several parameters: “skeletal muscle mass”, “appendicular skeletal muscle mass”, and muscle cross-sectional area at specific body sites. These parameters are corrected taking into account the body size using height, weight, body mass index, or body surface area. The muscle cross-sectional area is based on CT images at the level of the third lumbar vertebra (L3), where all muscles structures are contoured to define the skeletal muscle area (SMA) Are defined as sarcopenic the subjects with skeletal muscle index (SMI: SMA divided by body surface area) <52.4 cm^2^/m^2^ and <38.5 cm^2^/m^2^ in men and women, respectively.
What is the impact of sarcopenia in patients treated with radiotherapy?	Mainly from analyses on patients with head and neck tumors: ○ Significantly negative impact on overall survival. ○ Significantly worse toxicity and thus frequent radiotherapy breaks. ○ The negative impact results independent from other prognostic factors.
What is the impact of radiotherapy on sarcopenia?	Several side effects of radiotherapy (nausea, diarrhea, esophagitis, taste alterations, xerostomia) negatively impact the appetite and therefore lead to sarcopenia through reduced nutrition. Some evidence shows early weight reduction as independent of the onset of radiation-induced toxicity and of nutritional intake.
What is the impact of sarcopenic obesity and myosteatosis on radiotherapy outcomes?	Some studies reported that: ○ The negative impact of sarcopenia *plus* myosteatosis on overall survival is stronger compared to sarcopenia alone ○ Post-treatment sarcopenia and pre-treatment myosteatosis were independent predictors of reduced overall survival ○ Myosteatosis was independently associated with postoperative morbidity and worse overall survival in patients with retroperitoneal and trunk sarcoma treated with preoperative radiotherapy.
Can sarcopenia impact treatment modulation in patients who are candidates for radiotherapy?	The possibility of modulating radiotherapy based on the presence of sarcopenia would be very useful. However, there are currently no guidelines on the management of patients with sarcopenia treated with radiotherapy.
Can we improve the prognostic impact of sarcopenia by including it in predictive models?	The design of models including sarcopenia along with other prognostic factors seems an inevitable development of research on this topic. Only sporadic reports are available on this topic.
Is it possible to prevent sarcopenia before radiotherapy?	A pre-radiotherapy assessment of baseline levels would be desirable to identify early impairments or predisposing factors of the latter and to provide support to prevent subsequent deterioration of body composition and functional status. Unfortunately, this issue has been little studied.
Is it possible to treat sarcopenia before and during radiotherapy?	Two systematic reviews of the literature addressed this topic. ○ One study showed that the protein intake needed to avoid the loss of muscle mass during radiotherapy is higher than the usual recommendations. ○ One study showed that both nutritional interventions and exercise-based interventions, in patients undergoing radiotherapy for head and neck cancers are useful for maintaining nutritional status and physical functions while the combination of the two interventions is not effective.
Should we screen for and treat sarcopenia in radiation-treated pediatric patients too?	Only a few studies are available on this topic: ○ One study on a population of survivors of childhood acute lymphoblastic leukemia treated with hematopoietic stem cell transplant and total body irradiation showed lower insulin sensitivity, increased distribution of visceral, intramuscular, and total fat, and reduced lean body mass compared to leukemia patients treated only with chemotherapy. ○ A systematic review on body composition changes in patients 0 to 24 years of age undergoing the same treatments showed that a lipodystrophic phenotype and loss of skeletal muscle mass are common in these patients.
Which future studies on sarcopenia in the radiotherapy setting are needed?	Further studies are needed to: ○ Reach a broader consensus on definitions (and cut-offs) of sarcopenia, sarcopenic obesity, myosteatosis, and cachexia. ○ Define the best strategies for the prophylaxis and treatment of sarcopenia and compare them in randomized controlled trials. ○ Investigate on the effect of sarcopenic obesity on distribution, metabolism and elimination of chemotherapeutics used in concurrent chemoradiation. ○ Define methods of radiotherapy dose/fractionation-modulation in subjects with sarcopenia.

## Data Availability

Not applicable.
